# Ocular manifestations of Juvenile Systemic Lupus Erythematosus: a systematic review

**DOI:** 10.1038/s41433-025-03664-x

**Published:** 2025-02-17

**Authors:** Anna Nikolaidou, Theodora Gianni, Athanasia Sandali, Panagiotis Toumasis, Konstantinos Benekos, Efthymia Tsina

**Affiliations:** 1 Institute for Ophthalmic Research, University of Tuebingen, Tuebingen, Germany; 2 School of Medicine, Faculty of Health Sciences, Aristotle University of Thessaloniki, Thessaloniki, Greece; 3 Postgraduate Program MSc Ocular Surgery, School of Medicine, Faculty of Health Sciences, Aristotle University of Thessaloniki, Thessaloniki, Greece; 4 Ophthalmology Department, University of Ioannina, Ioannina, Greece; 5 Pediatric Ophthalmology Department, Athens Medical Centre, Athens, Greece

**Keywords:** Immunological disorders, Scientific community

## Abstract

Juvenile-onset Systemic Lupus Erythematosus (JSLE) is a chronic multifactorial autoimmune disease with multiple system involvement, affecting children and adolescents. Ocular manifestations are rare and can range from mild to severe. JSLE impacts quality of life and prognosis. However, the impact JSLE has on children’s ocular health remains an underexplored area. This systematic review aims to consolidate existing evidence on ocular manifestations in JSLE. A systematic search of MEDLINE and ScienceDirect was conducted until October 2024. Eligible studies focused on children and adolescents with JSLE presenting ocular symptoms. Joanna Briggs Institute critical appraisal tools for each study were employed for quality assessment. Forty-two studies evaluating ocular manifestations in JSLE patients were included. Among those were 29 case reports and case series with 34 patients in total, 4 cross-sectional studies, with a total of 210 patients, 155 of whom had ocular manifestations, and 9 cohort studies, with a total of 2696 patients enrolled and 212 of them reporting ocular manifestations (7.8%). Results were categorized by affected ocular structures: external, anterior, or posterior segment. Neuro-ophthalmological and drug-induced manifestations were described separately. Retinal involvement was the most often reported. Notable manifestations included dry eye disease, uveitis, vaso-occlusive retinopathy, and corticosteroid-induced cataracts. Ocular symptoms often served as the initial indication of disease onset. Heightened awareness and standardized assessments are necessary for management of ocular manifestations. Further research is needed to comprehensively elucidate the underlying mechanisms and pathogenesis of the disease.

## Introduction

Systemic lupus erythematosus (SLE) is a complex autoimmune rheumatic disease that affects various systems. It predominantly affects women of reproductive age, but this disease is not confined to adulthood. Roughly 15% to 20% of all cases arise during childhood and adolescence [1–3]. These cases constitute a separate clinical entity known as Juvenile-onset Systemic Lupus Erythematosus (JSLE) or childhood-onset Systemic Lupus Erythematosus (c-SLE) [4]. Despite similarities in clinical symptoms and laboratory findings between JSLE patients and adults, those with JSLE generally experience a higher incidence of major organ involvement and a more aggressive clinical course [5, 6]. JSLE, like adult SLE, demonstrates a racial distribution, where non-Caucasian groups exhibit a greater prevalence with manifestations at a younger age [7–11].

Even though ocular involvement is not part of the American College of Rheumatology diagnostic criteria for JSLE, JSLE can affect the eyes, with ocular involvement potentially stemming from either the inflammatory or thrombotic tendencies of the disease [12] and/or the immunosuppressant medications like glucocorticoids or antimalarial drugs, which are commonly prescribed to treat this disease [13]. More precisely, JSLE can be attributed to the mass, unregulated production of autoantibodies and immune complexes [14], which lead to intravascular activation of complement, inflammation, and tissue damage [15]. Ocular structures affected by this mechanism include the walls of blood vessels of the conjunctiva, sclera, ciliary body, choroid, and retina, as well as the basement membrane of the ciliary body and the epithelial basement [16]. It is noteworthy that almost one-third of the patients with JSLE may have ocular manifestations [17, 18], which, can range from mild to severe, potentially affecting the quality of life and long-term prognosis of affected individuals, and it has been reported that they may be presenting symptoms of the disease [19].

Although the systemic nature of JSLE is well-documented [20], its impact on children’s ocular health remains an underexplored area. There have been limited reports on ocular manifestations in JSLE [12, 13]. This systematic review aims to bridge this gap by synthesizing all the available evidence on ocular manifestations in JSLE, providing a foundation for a more comprehensive understanding of this aspect of the disease and providing clinicians with a reference point for assessing children with lupus.

## Materials and methods

This systematic review followed the guidelines outlined in the Preferred Reporting Items for Systematic Review and Meta-Analyses (PRISMA) 2020 statement [21] (Appendix A). All research procedures were carried out according to a protocol that was officially submitted in the PROSPERO database (Registration Number: CRD42023443580).

### Search strategy

An initial search method was created for the PubMed database until the 14^th^ of October 2024 including the following terms: “ocular manifestations”, “juvenile lupus erythematosus”, “childhood-onset lupus”, “paediatric lupus”, “ocular symptoms” and subsequently adapted for ScienceDirect using research articles and case reports as filters. The search string used for PubMed can be found in Appendix B. Reference lists of included studies were also screened manually. No restrictions were imposed on language or publication dates. The search process was carried out independently by two reviewers (AN and TG).

### Eligibility criteria

The eligible studies were those that implicated children and adolescents (age <18 years) with JSLE diagnoses presenting ocular symptoms. Only articles in the English language were included.

### Study selection

Studies were selected by two researchers (AN and TG) working independently after removing duplicates, and then title-abstract screened according to the inclusion criteria. Eligible studies were subsequently reviewed as full text by the researchers independently. A third researcher was not necessary since there was no discord between the researchers in the selection of studies.

### Data synthesis

We reported the results of the included studies according to the ocular structure that was affected as external (lacrimal system, orbit and eyelids), anterior segment (conjunctiva, cornea, sclera-episclera, uvea), posterior segment (retina, choroid), neuro-ophthalmological manifestations. Additionally, medication-related ocular events were noted. Retinal and neuro-ophthalmological manifestations were further subdivided. The pathophysiology of the ocular symptoms was extensively reported only for the retinal manifestations and was therefore further described. The percentage of JSLE cases with ocular manifestations was calculated for cohort studies.

### Quality assessment

The quality assessment of each included study was conducted independently by the two reviewers (AN and TG) using the appropriate Joanna Briggs Institute critical appraisal tool [22–25] depending on the type of study: for case reports, case studies, cross-sectional studies and cohort studies. Disagreements were resolved by discussion.

## Results

**Fig. 1:**
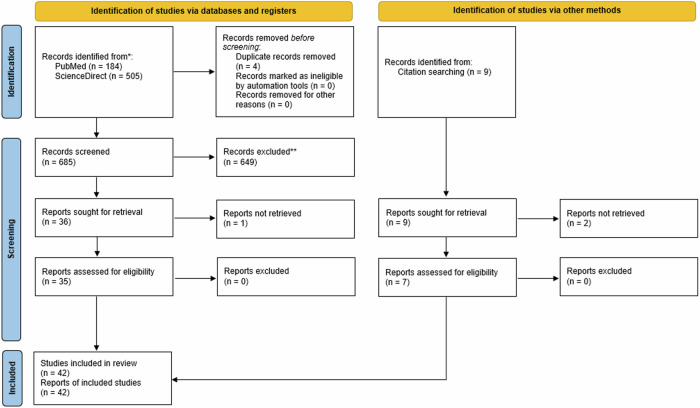
PRISMA flow diagram of the systematic review. The flow diagram illustrates the process of database and registry searches.

The initial search yielded 713 articles. Finally, forty-two eligible studies were analysed and appraised for methodological quality. The PRISMA flow diagram of the review process is depicted in Fig. 1. Reported manifestations of included studies are summarised and categorised in Table 1.

**Table 1:** Ocular manifestations in Juvenile Lupus Erythematosus.

*External*	
Lacrimal system: - Dry eye disease (Keratoconjunctivitis Sicca) [12, 18, 28, 31]	
Orbit: - Periorbital ecchymosis [32] - Orbital compartment syndrome [33] - Periorbital oedema [12, 35]	
Eyelids: - Upper eyelid skin involvement [12] - Eyelid oedema [36, 37] - Erythema and eyelid lesions [35]	

*Anterior Segment*	
Conjunctiva: - Follicular conjunctivitis [31] - Subconjunctival haemorrhage [38] - Chemosis/Injection [33, 35]	
Cornea: - Corneal vortex keratopathy [39] - Interstitial keratitis [40] - Superficial punctate keratitis [35]	
Sclera: - Anterior scleritis [35]	
Episclera: - Episcleritis [41] - Sectoral episcleritis [12]	
Uvea: - Anterior uveitis [18, 36, 42, 44] - Posterior uveitis [44] - Panuveitis [43, 44]	

*Posterior Segment*	
Retina: - JSLE retinopathy - Microangiopathy [18, 19, 51] - Vaso-occlusive Retinopathy [18, 19, 35, 38, 56, 58] - Retinal Vasculitis [18, 35, 42, 43, 45, 61] - Haemorrhagic retinopathy [46] - Hypertensive retinopathy [12] - Neuroretinitis [62]	
Choroid: - Chorioretinopathy [52] - Increased thickness [41] - Choroidal haemorrhages [44] - Choroidal ischaemia [42] - Choroidal detachment [35] - Chorioretinal scars [12]	

*Neuro-ophthalmological involvement*	
Optic nerve: - Optic neuritis [37, 67, 68] - Optic neuropathy [18] - Non-arteritic ischaemic optic neuropathy [55] - Papilledema [13, 31, 62, 69, 71] - Optic Atrophy [72, 74]	

*Medication adverse events*	
- Corticosteroid induced cataracts and glaucoma [13, 37, 44, 55, 61, 72, 76] - Hydroxychloroquine induced retinopathy [12, 13, 61, 78]	

Overall, 29 case reports and case series, 9 cohort studies and 4 cross-sectional studies were selected. A total of 2696 patients with JSLE were enroled in the cohort studies, with 212 of them reporting ocular manifestations (7.8%). We divided the included studies’ findings according to type of study (Tables 2–4). Of the 29 case studies and series reported, 34 JSLE patients had ocular manifestations, of which 15 were the inaugural manifestation of the disease (44%). Among these, 7 studies had concurrent systemic symptoms, while the remaining 8 reports solely described ocular manifestations as an initial presentation.

**Table 2:** Case reports and case series.

Study	Study Location	(*N*)	Gender	Age (y)	Ocular structure affected	Ocular Manifestations	Time of manifestation in disease course	Neuropsychiatric Involvement
Dorronsoro et al. [62]	Argentina	1	female	15	Retina	Bilateral neuroretinitis	Disease onset	No
Parakh et al. [42]	India	1	female	9	Retina, Uvea	Retinal vasculopathy uveitis	Disease onset	No
Jeon et al. [19]	Korea	1	female	13	Retina	Ischaemic vaso-occlusive retinopathy	Disease onset	No
Alhassan et al. [43]	USA	1	female	14	Retina	Bilateral retinal vasculitis, panuveitis	Disease onset	No
Lu et al. [78]	China	1	female	14	Retina	HCQ-induced retinopathy	7 years post-diagnosis	No
Deaner et al. [47]	USA	1	male	15	Retina	Vaso-occlusive retinopathy	1 year post-diagnosis	No
Huang et al. [54]	China	1	female	11	Retina	CRVO, CRAO	4 days post-diagnosis	No
Guleria et al. [51]	India	2	males	8 and 9	Retina	Retinal vasculopathy	Disease onset	Yes (encephalopathy, one patient)
Firl et al. [45]	USA	1	female	12	Retina	Retinal vasculitis	Disease onset	No
Abbas et al. [44]	USA	1	female	9	Cornea	Bilateral interstitial keratitis	6 months post-diagnosis	No
Jari et al. [32]	Iran	1	female	13	Orbit	Racoon eyes	Disease onset	Yes (hallucinations, aggressive behaviour)
Moreno Páramo et al. [56]	Mexico	1	male	14	Retina	CRAO & CRVO, vitreous haemorrhage	Disease onset	No
Hamill et al. [33]	USA	1	female	15	Orbit	Bilateral eyelid swelling	4 months post-diagnosis	Yes (cerebral oedema)
Fischer et al. [52]	Brazil	1	female	14	Choroid, retina	Bilateral chorioretinopathy	6 months post-diagnosis	No
Lanewalla et al. [71]	Pakistan	1	female	14	CNS, Optic nerve	Abducens nerve palsy causing strabismus and bilateral papilledema	3 months post-diagnosis	Yes (elevated ICP)
Georgakopoulos et al. [69]	Greece	1	female	14	Optic nerve	Bilateral Papilledema	Disease onset	Unclear (suspected VI nerve palsy)
Wei et el. [67]	USA	1	female	16	Optic nerve	Bilateral optic neuritis	2 years post-diagnosis	Yes (MRI: white lesions in the optic tract)
Chan et al. [31]	USA	1	female	8	Optic nerve, retina, conjunctiva, eyelids	Bilateral disc oedema, keratoconjunctivitis sicca, follicular conjunctivitis, symblepharon formation	Unclear	Yes (noncommunicating hydrocephalus secondary to stenosis of the aqueduct of Sylvius)
Ahmadieh et al. [68]	Iran	1	female	11	Optic nerve	Optic neuritis	3 months post-diagnosis	Unclear
Hackett et al. [70]	USA	1	female	11	Retina, Optic nerve	Optic neuritis, retinal oedema	Unclear	Yes (lethargy)
Suri et al. [37]	India	3	females	9, 14, 11	Optic nerve	C1: Unilateral optic neuritis C2: Unilateral Ischaemic optic neuropathy secondary to antiphospholipid syndrome. Sub-capsular cataracts with changes of glaucoma C3: Bilateral Optic neuritis	C1: Disease onset C2: 6 years post-diagnosis C3: Disease onset	Yes (C1: optic neuritis,brisk deep tendon reflexes, headache C2: optic neuritis, multifocal deep white matter lesions C3: optic nerve hyperintensities on STIR, optic neuritis)
Almeida et al. [36]	Brazil	2	females	15 and 20	Retina, uvea, lens, eyelids	C1: Iridocyclitis and severe retinal vasculitis with haemorrhage, bilateral eyelid oedema, irregular pupil and cataract C2:Bilateral retinal necrotizing vasculitis compatible with varicella zoster virus ocular infection	C1: 1-month post-diagnosis C2: 13 years post-diagnosis	Yes C1: acute confusional state C2: peripheral polyneuropathy
Zhang et al. [46]	China	1	female	15	Retina	Bilateral Hemorrhagic Retinopathy with Roth Spots. Sudden, painless vision loss unilaterally	1-year post-diagnosis	No
Nguyen et al. [35]	USA	1	female	16	Orbit, eyelids, conjunctiva, sclera, retina, macula, choroid	Periorbital oedema and erythema. Chemosis, conjunctival injection, diffuse scleritis, superficial punctate keratitis, flare, RPE detachment over the macula, retinal folds and elevation inferiorly, consistent with choroidal detachment	Disease onset	Not mentioned
Parchand et al. [58]	India	1	female	16	Rerina	Combined central retinal artery occlusion) and central retinal vein occlusion	6 months post-disease onset	Not mentioned
Graham et al. [38]	UK	1	female	16	Retina, conjunctiva	BRAO, deep retinal haemorrhage, subconjunctival haemorrhage	Disease onset	Yes (cerebral vasculitis and thrombosis)
Ho et al. [57]	Taiwan	1	female	16	Retina	Bilateral severe vaso-occlusive retinopathy	Disease onset	No
Palkar et al. [59]	India	1	female	15	Retina	Purtscher-like retinopathy	Disease onset	Not mentioned
Donnithorne et al. [55]	USA	2	females	13, 16	Retina	Retinal vasculitis and branch retinal artery occlusions	1-year and 1-month post-diagnosis	Not mentioned

**Table 3:** Cross-sectional studies.

Study	Study Location	Total (*N*)	Gender (male)	Age (years)	Ocular JSLE (*N*)	Ocular structure affected	Ocular Manifestations
Paim-Marques et al. [39]	Brazil	76	15	Mean: 17.9 (SD ± 3.07)	76	Cornea	Corneal vortex keratopathy
Ağın et al. [41]	Turkey	42	Unclear, gender-matched	Median: 16 (6 to 19)	21	Episclera, Lens	Episcleritis (1 patient) Corticosteroid-induced cataract (1 patient)
Gawdat et al. [12]	Egypt	40	8	Mean: 13	40	Retina, Lens, Conjuctiva, Orbit, Episclera	Ocular hyperaemia (4 patients), periorbital oedema (one patient), photosensitivity (24 patients)
Al-Mayouf et al. [18]	Saudi Arabia	52	7	Mean: 11.3	18	Conjunctiva, lens, optic nerve, retina, uvea	18 patients with ocular manifestations: 7 with abnormal Schirmer’s test (2 bilateral, 5 unilateral). 5 with retinal vascular lesions (4 unilateral, one bilateral). 1 had bilateral iridocyclitis. 3 with unilateral optic neuropathy and 11 patients with visual field defects (4 bilateral, 7 unilateral)

**Table 4:** Cohort studies.

Study	Study Location	JSLE Total (*N*)	Gender (male)	Age (years)	Follow-up duration (years)	Ocular JSLE (*N*)	Ocular JSLE (%)	Ocular Manifestations
Lim et al. [75]	Malaysia	141	17	Median:10.8 (9 to 12)	6.3 (range 3.6–9.0)	42	29.7%	Ocular damage was the most common side effect (29%) and was predominantly corticosteroid related (93%). Cataract (29/42), Glaucoma (10/42)
Tone et al. [28]	Canada	34	4 with JSLE, 5 healthy	Mean: 15.4 (2.1)	Unclear	Unclear	20.6% to 58.8% (depending on the ophthalmologic assessment test performed)	Dry eye disease
Kahwage et al. [44]	Brazil	852	NA	≤ 25 (current age at onset of uveitis 8–14)	2	7	1%	Uveitis (7/852, 0.8%). Cataract and irreversible blindness (1/852) Retinal ischaemia with neovascularization and unilateral blindness (1/852)
Koutsonikoli et al. [72]	Greece	47	8	Mean: 12.5 (3.1)	Unclear	7	14%	Ocular tissues were the most frequently affected. Cataract (4 patients) - Retinopathy (1 patient) - Optic atrophy (1 patient) - Cataract & Retinopathy (1 patient)
Fraga et al. [13]	Brazil	117	17	Mean: 10.4 (NA)	5.4	24	20.5%	Abnormal fundoscopy associated with systemic hypertension and/or use of chloroquine (16/117), Cataract (4/117), Glaucoma (2/117), Cataract & Glaucoma (2/117)
Salah et al. [73]	Egypt	148	45	Mean: 17.1 (3.8)	6.57 ± 3.59	9	6.1%	Cataract: 3,4% Retinal change or optic atrophy: 2,7%
Chan et al. [76]	Taiwan	904	130	ΝΑ	5	79	10.7%	Cataracts and glaucoma
Ravelli et al. [74]	Europe, US, Mexico, and Japan (multi-centre)	387	57	Mean: 17.1 (5.4)	5 ( ± 3.6)	42	10.9%	Any cataract 7.5% Retinal change or optic atrophy 4.4%
Taddio et al. [61]	Italy and US	100	21	Mean: 12.7 (3.1)	5.3 ( ± 2.3)	2	5%	Retinal vasculitis

Across studies, different indexes were used for disease severity including the Systemic Lupus Erythematosus Disease Activity Index (SLEDAI) [26] and the Systemic Lupus International Collaborating Clinics (SLICC) [27] criteria. These indices serve as tools in the clinical evaluation and classification of SLE and encompass various clinical and laboratory parameters to assess disease activity. SLICC criteria offer a comprehensive framework for the classification of lupus, considering diverse clinical manifestations over time.

### External

#### Lacrimal system

Dry eye disease is the most common ocular manifestation of SLE among adults. Dry eye in JSLE could be the result of inflammation of the ocular surface, tear film and/or meibomian glands [28–30]. In our review, one case report [31] and three studies (a cross-sectional study of 40 patients total [12], a cohort study of 49 participants total [34 with JSLE [28]] and a cross-sectional study of 52 patients total [18] reported dry eye disease in JSLE patients.

In their descriptive cross-sectional study involving 40 patients, Gawdat et al. [12] found an abnormal Schirmer test in 16 of them (40%), leading to the identification of keratoconjunctivitis sicca (dry eye syndrome) as the predominant JSLE manifestation. However, the authors hypothesized that the dryness could also be attributed to the systemic medications used, other than the disease pathology itself. Furthermore, 24 (60%) patients in this study mention photophobia as their first symptom. Tone et al. [28], in their prospective, observational, single-centre cohort study reported that 34 children with SLE exhibited elevated average corneal fluorescein scores compared to healthy children (1.7 ± 1.7 vs. 0.2 ± 0.4; *p* = 0.002), increased combined mean scores for corneal and conjunctival staining, and a greater proportion of abnormal corneal fluorescein scores in comparison to healthy children (58.8% vs. 20.0%; *p* = 0.01), showing possible dry ocular surface signs associated with the disease. This study, however, only showed a higher incidence of dry eye signs in JSLE patients, rather than a true ocular manifestation. Al-Mayouf et al. [18] found that seven out of fifty-two JSLE patients had an abnormal Schirmer test, with no association with Sjögren’s syndrome and only two of them demonstrated bilateral involvement. The authors note that in JSLE patients, varied degrees of keratoconjunctivitis sicca might be combined with xerostomia, episcleritis, interstitial keratitis, periorbital oedema, and eye movement abnormalities. Chan et al. [31] presented a case report of an 8-year-old female patient with JSLE who had a history of chronic eyelid oedema and ocular itching. The patient presented with ocular surface pathology, including keratoconjunctivitis sicca with mucus deficiency.

#### Orbit

Orbital involvement is rare among JSLE patients. In our review, six studies in total mention ocular findings on the orbit. These findings include mostly periorbital oedema and ecchymosis which could be attributed to increased vascular permeability, iridocyclitis, renal dysfunction or, rarely, orbital compartment syndrome associated with systemic vasculitis [17].

Jari et al. [32] describe the case of a 13-year-old girl presenting for the first time with swelling and periorbital ecchymosis (“raccoon eyes”), which was followed by blurred vision in her left eye. Additionally, she exhibited neuropsychiatric manifestations, including hallucinations and aggressive behaviour. The periorbital swelling was postulated to be the result of increased vascular permeability. Another case report [33] describes a 15-year-old female with bilateral progressing periorbital oedema, a bilateral tight orbit, pulsatile proptosis, unilateral lagophthalmos with conjunctival chemosis, and intraocular pressure of 82 mmHg in the eye with the lagophthalmos and 54 mmHg in the other one. These were attributed to orbital compartment syndrome in the setting of diffuse multiorgan oedema and evolving cerebellar herniation, which were thought to be related to systemic vasculitis. Despite surgical and pharmaceutical interventions, the patient passed away. She had severe cerebral oedema secondary to Neuropsychiatric SLE. Adult patients with Neuropsychiatric SLE have been reported to have a mortality rate 10 times higher than that of healthy subjects [34]. Gawdat et al. [12] reported that one patient exhibited periorbital oedema as a presenting symptom of JSLE, which was suggested to be indicative of renal involvement. Nguyen et al. [35] also reported a 16-year-old female who first presented with periorbital oedema, painful ocular motility, and decreased vision unilaterally, demonstrating inflammatory involvement of multiple ocular structures.

#### Eyelids

Gawdat et al. [12] recorded three JSLE patients that exhibited ocular manifestations involving the upper eyelids; one displayed diffuse redness, another showed darkened skin areas, and the third presented discoid skin eruptions. The study found that none of these manifestations demonstrated a correlation with the disease duration and/or the SLEDAI-2K score. Almeida et al. [36] mention bilateral eyelid oedema in the context of iridocyclitis in a 15-year-old female among a case series of 263 patients. In the case report by Nguyen et al. [35], the 16-year-old girl exhibited erythema and scattered papular, erythematous lesions on the eyelids unilaterally and on the cheek. Suri et al. [37] identified a case of bilateral eyelid oedema, referred to a 9-year-old girl with a normal orbit MRI but concomitant renal involvement.

### Anterior segment

#### Conjunctiva

Chan et al. [31] described an 8-year-old female diagnosed with JSLE with a moderate follicular response of the conjunctiva, a true membrane and symblepharon formation in the inferior conjunctiva, pointing to keratoconjunctivitis sicca and follicular conjunctivitis. This was suspected to be the result of polyclonal B-cell activation, autoantibody production and intravascular activation of complement, leading to tissue damage. Graham et al. [38] presented a 16-year-old male with subconjunctival haemorrhage and unilateral mild visual impairment. Examination revealed vaso-occlusive retinopathy. Unfortunately, his death occurred twelve weeks later due to left ventricular failure secondary to hypertension and pulmonary embolism. Conjunctival involvement is also reported as a concurrent condition in the form of chemosis and/or conjunctival injection in cases of periorbital oedema reported in JSLE patients [33, 35].

#### Cornea

Three studies reported clinical manifestations in the cornea, which included corneal vortex keratopathy [39], interstitial keratitis [40] and superficial punctate keratitis [35]. Paim-Marques et al. [39] reported 36 patients with ocular abnormalities in a cohort of 76 JSLE patients, including 16 with corneal vortex keratopathy. All patients with keratopathy were females on chloroquine. Nguyen et al. [35] also described superficial punctate keratitis in a 16-year-old female who presented with periorbital oedema and signs of panuveitis. Abbas et al. [40] reported a case of bilateral interstitial keratitis in a 9-year-old African American female as an inaugural manifestation of JSLE. Slit lamp examination revealed bilateral superficial and deep peripheral corneal stromal opacification, extending from peripheral cornea to the centre, combined with vascular loops in the corneal stroma. After 6 months of immunosuppressive topical and systematic treatment, peripheral corneal scarring remained but stromal inflammation was limited, and corneal vascular loops regressed. Over a 12-year follow-up, no other ocular manifestations were reported, suggesting JSLE as the primary cause of the corneal involvement.

#### Sclera—episclera

Reports in the literature suggest that scleritis and episcleritis may occur as rare ocular manifestations in JSLE; in a single-centre, cross-sectional study from 2019 involving 21 patients with JSLE, Ağın et al. [41] recorded a case of episcleritis. In another descriptive cross-sectional study [12] with 40 patients in total, sectoral episcleritis was identified in one patient.

The patient described by Nguyen et al. [35] presented with diffuse scleritis alongside periorbital oedema and erythema. The patient also exhibited signs of anterior uveitis as well as choroidal detachment and partial retinal pigment epithelium (RPE) detachment. B-scan ultrasonography showed both anterior and posterior scleritis in one eye and mild posterior scleritis in the other. Symptoms improved as she was treated with corticosteroids and azathioprine.

#### Uvea

The cross-sectional study with 52 JSLE patients by Al-Mayouf et al. [18] includes a case of bilateral iridocyclitis, which coincided with a flare of systemic disease. Another case of severe uveitis with retinal vasculitis and cataracts is presented in a case series by Almeida et al. [36], causing a drop of visual acuity lower than 20/200. The 15-year-old female patient exhibited bilateral anterior chamber severe inflammatory reaction with hypopyon, irregular pupils and periorbital oedema. The drop in visual acuity remained unchanged despite treatment with immunosuppression and the patient remained legally blind.

Parakh et al. [42] reported a case of bilateral mild anterior uveitis along with retinal vasculopathy in a 9-year-old female who presented with gradually decreased vision bilaterally, accompanied by pain and redness. Alhassan et al. [43] also present a case of panuveitis in a 14-year-old girl, which constituted as the disease’s first and only presentation along with retinal vasculitis. Nguyen et al. [35] also reported inflammation of the anterior chamber in a case of a 16-year-old with JSLE choroidopathy.

A retrospective multicentre cohort study by Kahwage et al. [44] reported that among 852 patients with JSLE, 7 (0.8%) presented uveitis, four of which had posterior uveitis and one had panuveitis. The average age of onset was 10 (range 8–14) years. Uveitis was identified at the time of JSLE diagnosis in four patients (57%), within the first 6 months of disease in two patients, and after 31 months of follow-up in one patient. One patient exhibited uveitis with retinal ischaemia and subsequent neovascularization resulting in unilateral blindness. Patients with uveitis had an increased SLEDAI-2K score (19 vs. 6; *p* < 0.01). Posterior uveitis was more frequently detected, even though neither the anterior nor posterior segments of the eye were shown to be more susceptible to lupus.

### Posterior segment

#### Retina

Twenty-one studies reported clinical manifestations from the retina, constituting the most common ocular structure affected that was reported in our review. Among the retinal manifestations, SLE retinopathy and retinal vasculitis are correlated to the disease itself and its inflammatory and occlusive pathogenesis. Additionally, neuroretinitis, haemorrhagic retinopathy and hypertensive retinopathy are indirectly correlated to the disease through mechanisms of inflammation, thrombocytopenia, and hypertension respectively.

##### JSLE retinopathy

Retinal findings in JSLE primarily stem from antiphospholipid antibodies and immunoglobulin accumulation causing luminal narrowing, active vasculitis triggering inflammatory pathways and thrombosis [45], thrombocytopenia [46] and systemic hypertension vasculopathy [12] and immunosuppression vulnerability of ocular infections [36]. One theory suggests that during lupus active vasculitis, there is a disruption of the vascular endothelium, exposing phospholipids that can potentially act as antigenic triggers, leading to the production of anti-phospholipid antibodies, or they may become targets for circulating anti-phospholipid antibodies [47, 48].

Vascular thrombosis is also thought to be attributed to antiphospholipid syndrome as an immune complex-mediated vasculopathy rather than a being a true vasculitis associated with JSLE itself [49]. While the primary causes of retinal involvement in JSLE are focused on autoimmune processes associated with vasculopathy, the exact mechanisms are yet to be clarified. Retinal involvement in systemic lupus erythematosus often correlates with systemic disease activity and possible progression [50] while it varies in severity and can occur at any stage of the disease. It has also been shown in adults that vaso-occlusive retinopathy strongly correlates Central Nervous System (CNS) involvement [49].

However, in our review, children with vaso-occlusive retinopathy had no neurological symptoms except for one with generalized tonic-clonic seizures and altered sensorium [51]. Renal involvement has also been shown to be related to retinal involvement [52, 53]. Cases in our review reported severe renal involvement presenting several months after retinal manifestations [52] or at diagnosis alongside retinal involvement [38]. SLE retinopathy is characterized by microangiopathy, involving widespread non-perfusion of capillaries in the retina, along with occlusions occurring in small or bigger vessels.

SLE retinopathy pathogenesis stems from the occlusion of retinal vessels, primarily caused by the deposition of circulating immune complexes. This process results in fibrinoid degeneration and necrosis of the vessel wall rather than inflammatory vasculitis [51]. The condition predominantly affects small vasculature, leading to microangiopathy, with clinical presentations including cotton-wool spots and perivascular exudates, retinal haemorrhages, or microaneurysms. The elasticity of the vascular wall in children and abundant microcirculation may also act as indicators of better prognosis than in adults [54]. Larger vessels such as the central retinal artery may be occluded, resulting in sudden painless vision loss, while involvement of a branch of the retinal artery can cause visual field defects. If left untreated, those findings can lead to significant irreversible visual impairment. Vaso-occlusive retinopathy and retinal vasculitis are part of the same spectrum of vascular disturbances in SLE. They can co-exist and also manifest independently. Vasculitis can potentially lead to severe vessel occlusion due to immune complexes deposition.

*i. Microangiopathy*: Guleria et al. [51] reported 2 cases of paediatric lupus retinal vasculopathy in 2 school-age males. Both patients in this series had cotton-wool spots and showed occlusive microangiopathy, both of which, along with systemic symptoms, were present at disease onset. Ischaemic vaso-occlusive retinopathy as the inaugural symptom of JSLE is showcased by Jeon et al. [19] describing a 13-year-old female, with bilateral perivascular haemorrhages around ghost vessels and macular oedema. She was treated with hydroxychloroquine and steroids, vitreous injection of triamcinolone in one eye and dexamethasone intravitreal implant insertion to the other. After no signs of improvement, panretinal photocoagulation (PRP) was performed, which lessened her clinical symptoms. In a study by Al-Mayouf et al. [18] including 52 JSLE patients, one case was reported with retinal vascular lesions in the context of vaso-occlusive disease.

*ii. Vaso-occlusive retinopathy*: Deaner et al. [47] described a 15-year-old female with unilateral vaso-occlusive retinopathy attributed to antiphospholipid syndrome associated with JSLE one year post-diagnosis. The patient was treated with immunosuppression and anti-coagulation agents and showed evidence of vascular reperfusion, implying the reversible nature of vaso-occlusive disease. Donnithorne et al. [55] describe two cases with signs of branch retinal artery occlusions in two teenage females combined with retinal vasculitis, one of which had non-arteritic ischaemic optic neuropathy as well. One year after JSLE diagnosis, she developed branch retinal artery occlusion with macular ischaemia, retinal neovascularization, vitreous haemorrhage, and tractional retinal detachment for which she received PRP treatment leading to slight improvement of her vision. The second case was diagnosed with JSLE a month before she exhibited retinal vasculitis, showing fundus signs of vasculitic changes in the macula, intraretinal haemorrhages and cotton wool spots. She was treated with immunosuppressants and hydroxychloroquine. Vasculitis almost completely resolved unilaterally, whereas vision was significantly improved bilaterally.

Most case reports documented vaso-occlusive retinopathy being the only, or part of the first manifestations of JSLE. Huang et al. [54] described unilateral vaso-occlusive retinopathy being part of the first flare-up of the disease in an 11-year-old female with central retinal vein and artery occlusion with diffuse retinal haemorrhages and macular oedema that was treated with steroids, PRP, and intravitreal injection of dexamethasone with partial vision improvement. Paramo et al. [56] reported a unilateral central retinal vein and artery occlusion as the inaugural manifestation of JSLE in a 14-year-old male who also developed vitreous haemorrhage which had been treated with intravitreal antiangiogenics and immunosuppressants. Another vaso-occlusive retinopathy case as the initial ocular presentation of JSLE, was presented by Ho et al. [57]. A 16-year-old female fundus examination revealed bilateral vaso-occlusive retinopathy with macular “cherry-red spot” appearance, multiple confluent cotton wool spots, and widespread arterial occlusion, alongside with other systemic manifestations of JSLE. Steroid treatment only improved vision in one eye, and later bilateral PRP treatment was performed due to neovascularization, which only improved vision in one eye. However, the other eye developed tractional retinal detachment, which eventually led to visual acuity of hand movement.

Graham et al. [38] described a 16-year-old female with a cotton wool spot and deep retinal haemorrhage in the right eye, while the left eye exhibited occlusion of the infero-temporal retinal artery, leading to extensive retinal swelling. The patient had presented with subconjunctival haemorrhage and unilateral mild visual impairment. She underwent treatment with prednisolone and azathioprine but finally succumbed to left ventricular failure secondary to hypertension and pulmonary embolism. Post-mortem examination revealed systemic complications of severe acute JSLE, including renal failure, cerebral vasculitis, and non-inflammatory vascular disease in the retina. Retinal arteriolar occlusion was attributed to either embolism from a diseased heart valve or local antigen-antibody complex formation. Post-mortem findings included occasional vessel occlusion with hyaline thrombus in the retina, deep haemorrhage, nerve-fibre swelling, and choroidal inflammation with arteriolar occlusion and vasculitis. Parchand et al. [58] showcased the variety of retinopathy in JSLE, from isolated mild retinopathy with cotton wool spots to severe occlusive retinopathy with large vascular occlusions including central retinal artery and vein occlusion, which were described in 16-year-old female. Corticosteroids, anticoagulants, and immunosuppressive agents were administered, while PRP in the eye with severe retinopathy were performed. Despite those efforts, she developed a vitreous haemorrhage on follow-up.

Purtscher-like retinopathy, a severe form of JSLE retinopathy, has been reported as presenting manifestation of JSLE alongside systemic manifestations by Palkar et al. [59] in a case report of a 15-year-old female. The patient showed retinal whitening with non-perfusion in fluorescein angiography, along with optic disc oedema, marked venous tortuosity, intraretinal haemorrhages, and cotton wool spots unilaterally, all of which were improved after treatment with immunosuppressants and hydroxychloroquine.

*iii. Retinal Vasculitis*: Four case reports [42, 43, 45, 60] recorded retinal vasculitis as the inaugural manifestation of JSLE. Almeida et al. [36] described two young females, both legally blind due to vasculitis, reported among 263 patients with JSLE. In the first case, a 13-year-old exhibited severe retinal vasculitis with haemorrhage, bilateral eyelid oedema, irregular pupils and cataract due to uveitis 1 month after JSLE diagnosis which caused a severe vision impairment that was not improved with immunosuppressive treatment. The second case, diagnosed with JSLE 13 years before, exhibited bilateral retinal necrotizing vasculitis compatible with varicella zoster virus ocular infection causing severe visual impairment. She was unsuccessfully treated with antiviral medication. Both cases were on hydroxychloroquine for different periods of time, and both had concurrent CNS involvement, with confusional mental state and polyneuropathy respectively.

Alhassan et al. [43] reported a case of a 14-year-old female with JSLE, presenting with bilateral retinal vasculitis and panuveitis. Bilateral diffuse haemorrhages, white retinal lesions, and blurred optic disc margins were evident and treated with immunosuppressants. Firl et al. [45] also reported retinal vasculitis as the inaugural symptom of JSLE in a 12-year-old female with bilateral diffuse intraretinal haemorrhages and retinal neovascularization associated with macular traction and striae. Extensive peripheral vascular sheathing and retinal capillary nonperfusion were also evident bilaterally but there was no information about the visual outcome. Nguyen et al. [35] describe a case of retinal vasculitis three days post-admission for choroidopathy in a 16-year-old female. Clinical findings pointed towards the overriding problem being autoimmune vasculitis, likely an Overlap Syndrome. She received high dose intravenous pulse cyclophosphamide that led to regression of symptoms. Parakh et al. [42] report a case of 3-month diminution of vision which was attributed to bilateral extensive retinal vasculitis with macular oedema. The patient also exhibited systemic symptoms which responded well to intense systemic immunosuppression along with topical steroids and PRP. Taddio et al. [61] performed a multicentre retrospective cohort study of 100 patients with JSLE, of which two exhibited retinal vasculitis.

##### Haemorrhagic retinopathy

Zhang et al. [46] described a case of a 15-year-old female with bilateral haemorrhagic retinopathy with Roth spots, who experienced sudden painless loss of vision unilaterally. The patient had been thrombocytopenic for a year and had stopped medication 3 months before. Subretinal haemorrhages around the optic disc were identified. The manifestation was attributed to thrombocytopenia that causes disruption of the vascular integrity and haemostasis.

##### Hypertensive retinopathy

Gawdat et al [12] describe 7 cases of retinal vascular changes among 40 children with JSLE with fundoscopic findings of arteriovenous crossing in two, arterial attenuation in three and congested tortuous veins in two patients. All patients had systemic hypertension and were on medication. The retinal vascular changes were linked to hypertension associated with JSLE and could not be correlated with disease duration or activity. Fraga et al. [13] reported twelve out of twenty-four JSLE patients with systemic arterial hypertension, of which three (25%) had cotton wool spots and papilledema.

##### Neuroretinitis

We identified only one case of neuroretinitis as an ocular manifestation of JSLE. Dorronsoro et al. [62] presented a case of a 15-year-old female introducing a rare association of concurrent neuroretinitis and membranous lupus nephritis at disease onset. The adolescent had bilateral papilledema with partial macular star and a mild visual impairment. Treatment with hydroxychloroquine benefited the clinical image with visual improvement to 20/20 and disappearance of macular exudates and papilledema through a time course of 7 months.

#### Choroid

Ağın et al. [41] theorize that JSLE may affect the choroid; the authors used spectral domain Optical Coherence Tomography to measure choroidal thickness, finding that in JSLE patients the choroid was significantly thicker compared to the control group. Measurements of the total subfoveal choroidal area (TCA), the luminal area and the stromal area were also found to be higher than the control group. Several studies may support this finding; Fischer et al. [52] describe a case of bilateral chorioretinopathy with transitory visual impairment in a 14-year-old female after 6-months of JSLE diagnosis. Four cases of choroidal haemorrhages due to posterior uveitis were also observed in a retrospective multicentre cohort study by Kahwage et al. [44] which included a total of 852 patients. Additionally, Parakh et al. [42] described peripapillary areas of greying discovered during examination, suggestive of choroidal ischaemia, in a 9-year-old female with JSLE retinopathy.

Nguyen et al. [35] present a case of JSLE with anterior segment inflammation and unilateral unveiled retinal folds and elevation, pointing to choroidal detachment, as well as RPE detachment over the macula. The 16-year-old female patient presented with periorbital oedema, painful ocular motility, and unilateral decrease of vision. She then developed retinal vasculitis and unilateral exudative retinal detachment which significantly improved after immunosuppressive treatment. In the descriptive cross-sectional study involving 40 JSLE patients, Gawdat et al. [12] detected a case of a 13-year-old JSLE patient with multiple bilateral pigmented chorioretinal scars and concurrent neurological symptomatology. The authors attributed the chorioretinal scars to a possible previous attack of chorioretinitis associated with neurological JSLE.

### Neuro-ophthalmological manifestations

Fourteen studies reported clinical manifestations in the optic nerve, such as optic neuritis or neuropathy, optic atrophy or papilledema, all described in teenage females. The pathogenesis of optic neuritis in JSLE, as in adult SLE, primarily involves vaso-occlusion of small vessels, leading to subsequent demyelination [63]. In cases exhibiting pure demyelination, research suggests that demyelination might result from a less severe form of ischaemic injury affecting small vessels [64]. However, in more severe cases, there’s evidence of axonal damage and necrosis alongside demyelination [65]. Both unilateral and bilateral cases of optic neuropathy have been reported with bilateral being more frequent, according to a study conducted in adults [66]. Unilateral disease is most likely caused by a focal thrombotic event that should be treated with anticoagulation, while bilateral involvement is suggested to be the result of systemic vasculitic process and should be treated with immunosuppression [67, 68]. Optic nerve involvement, when reported as papilledema, is usually combined with retinal changes in the context of intracranial hypertension.

#### Optic neuritis/optic neuropathy

Suri et al. [37] describe two cases of optic neuritis as the first presentation of JSLE, both manifesting as vision impairment, bilaterally and unilaterally in an 11-year-old and a 9-year-old, respectively. A third case presented in the same study, mentions optic neuropathy secondary to antiphospholipid syndrome, combined with posterior subcapsular cataracts with changes of glaucoma due to corticosteroid medication in a 14-year-old female. In all cases, there was CNS involvement, including headache, brisk reflexes, MRI findings of hyperintensities, white matter lesions and possible encephalomyelitis. Ahmadieh et al. [68] reported an 11-year-old female with predominantly retrobulbar optic neuritis, optic disc swelling and involvement of optic nerve head, who had multiple attacks of neuritis, starting 3-months post-diagnosis. Wei et al. [67] also presented a 16-year-old female patient who experienced bilateral retrobulbar optic neuropathy 2 years after being diagnosed. The patient was seropositive for both antiphospholipid and neuromyelitis optica antibodies and had JSLE complicated with nephritis. Both cases were treated with high doses of corticosteroids and immunosuppression. Al-Mayouf et al. [18] recorded three patients with unilateral optic neuropathy in their cross-sectional study of 52 patients, where a significant positive correlation was found between optic neuropathy and CNS involvement. Donnithorne et al. [55] also present a case of a 16-year-old female showing evidence of unilateral non-arteritic ischaemic optic neuropathy, which eventually caused an afferent pupillary defect in the left eye.

#### Papilledema

Six studies mention papilledema in children with JSLE, all of them reporting concurrent CNS involvement. In a 12-year retrospective cohort study from Brazil involving 117 patients, Fraga et al. [13] describe papilledema in three patients concurrent with arterial hypertension, while Georgakopoulos et al. [69] mention another case of bilateral papilledema in a 14-year-old female, attributed to intracranial hypertension secondary to JSLE. Dorronsoro et al. [62], describe a 15-year-old female showing bilateral papilla oedema with partial macular star more evident unilaterally, as a result of bilateral neuroretinitis, alongside with systemic symptoms at disease presentation. Neuroretinitis resolved after 7 months of immunosuppressive treatment.

Cases of bilateral disc oedema are also presented by Chan et al. [31] and Hackett et al. [70]. Chan et al. [31] report a case of an 8-year-old female, where funduscopic evaluation revealed bilateral disc oedema with absent spontaneous venous pulsation. The patient’s history included noncommunicating hydrocephalus secondary to stenosis of the aqueduct of Sylvius. Hackett et al. [70] describe an 11-year-old female, who later passed away, with optic neuritis presenting with ptosis, retrobulbar pain and loss of vision bilaterally, with progressive disc oedema and flame haemorrhages, congested veins, and retinal oedema.

Lanewalla et al. [71] mentioned a case of bilateral papilledema and abducens nerve palsy in a 14-year-old female, also causing strabismus and acute-onset diplopia. These were suspected to be the result of lupus cerebritis and of raised intracranial pressure. Drainage of cerebrospinal fluid, as opposed to immunosuppression, proved beneficial.

#### Optic atrophy

In a retrospective cohort study that evaluated data from a 25-year period in Greece [72], one patient out of 47 JSLE patients developed optic atrophy. Optic atrophy was also mentioned to be a sign of JSLE ocular damage in a retrospective study in Egypt [73], as well as in a multi-centre cohort study by Ravelli et al [74]. They reported 387 cases of JSLE, of which 10.9% had ocular involvement, including 4.4% had retinal changes or optic atrophy. Similarly, Salah et al. [73] showed that among 148 patients, 6.1% had ocular involvement, of which 2.7% of retinal change or optic atrophy.

### Medication ocular adverse effects

#### Corticosteroids

Specific ocular manifestations often arise as a consequence of medication administered to patients with JSLE. The most prevalent of these adverse side effects are cataract formation and glaucoma, primarily attributed to high doses of corticosteroids, which are frequently employed as the first line of treatment.

Ağın et al. [41] in their single-centre cross-sectional study reported one JSLE patient out of twenty-one, with corticosteroid-induced cataracts. Gawdat et al. [12] also mentioned one patient with a faint posterior subcapsular cataract, who had been on systemic steroids for 6 years. In their cross-sectional study, Al-Mayouf et al. [18] included 4 out of a total of 52 patients who developed posterior subcapsular cataracts. Most patients had JSLE for at least one year and were receiving prednisone.

The study by Fraga et al. [13] mentioned eight patients, who had been using corticosteroids for a period ranging from 10 to 84 months, that developed cataract and/or glaucoma; four cases of isolated cataract, two cases of both cataract and glaucoma and two cases of isolated glaucoma. Sixteen patients had fundoscopic abnormalities such as cotton wool spots, papilledema, and macular changes, which were associated with systemic hypertension and/or the use of chloroquine. A statistically higher and longer dosage of steroids was administered to patients that eventually developed ocular changes, while all 24 patients with ocular manifestations were on glucocorticoids and chloroquine.

In a 12-year retrospective cohort study [75] involving 141 JSLE patients, 42 had ocular manifestations. The eye was the most affected organ (29%) with almost all of the manifestations (93%) being related to side effects of corticosteroid treatment. Ocular damage, including 29 cases of cataracts and 10 cases of glaucoma, occurred early and ascended with time. It was mentioned that the study could be biased towards severe cases of JSLE.

Among large cohort studies that were included, many sparsely reported cases of cataract and glaucoma were evident as ocular involvement without a clear relation to treatment modalities or duration. Kahwage et al. [44] mentioned one patient, described in detail by Donnithorne et al. [55], with cataracts and irreversible blindness due to ischaemic optic neuropathy. Suri et al. [37] also presented a case of bilateral subcapsular cataract with changes of glaucoma in a 14-year-old patient on prednisolone, steroid-sparing drugs, azathioprine, and hydroxychloroquine. In the study by Koutsonikoli et al. [72], five cases of cataracts with one case of concurrent retinopathy were recorded.

Salah et al. [73] observed cataracts in 3.4% of their 148 JSLE patients. Similarly, Chan et al. [76] mentioned cases of cataracts and glaucoma in a study where ocular manifestations constituted a percentage of 10.73% in 904 patients. In comparison to the general public, cataracts and glaucoma were recorded in a higher percentage of children (2.77% versus 0.15% and 5.97% versus 0.66% respectively). Taddio et al. [61] reported ocular complications as the most common, in 10 patients out of 100, 7 with cataracts and 3 with retinal change. All of them were on steroid treatment. Moreover, 7.5% of patients in the study Ravelli et al. [74] developed some form of cataract.

#### Hydroxychloroquine/chloroquine

Hydroxychloroquine (HCQ), commonly used in JSLE, has been associated with retinal damage, recognised as HCQ-induced retinopathy. The mechanism behind this adverse reaction is currently unclear, but it is theorized that HCQ or its byproducts are accumulated in the retina, due to the molecules’ strong ability to bind to the pigment tissue of the eye [66]. HCQ affects the ganglion and photoreceptor cells at first, and as a result both the choroid and the RPE eventually deteriorate [77].

Lu et al. [78] described the case of a 14-year-old female with JSLE for 7 years, who had been using a safe dose of 2.6 mg/kg/day HCQ for 20 months and presented with rapidly decreasing vision in the left eye over 3 days. Her vision was 20/20 in both eyes with a stable local relative visual field defect. Symptoms were attributed to HCQ retinal toxicity, which is thought to be related to kidney disease [79], as the patient had severe JSLE nephritis and therefore lower safety threshold.

Gawdat et al. [12] found RPE atrophic changes in five patients, scattered in the fundus in three cases and localized in the paramacular area in two, documented as RPE mottling. All five patients had used or had been using HCQ for a duration between 6 months and 10 years. Fraga et al. [13] mentioned that 8 JSLE patients of 16 with abnormal fundoscopy had macular changes, related to chloroquine use ranging between 1 and 5 years. Three of those patients had been on HCQ, two on chloroquine diphosphate, and three on chloroquine diphosphate and HCQ afterwards. In three patients, treatment had to be discontinued. The mean age of presentation of ocular manifestations was 14.6 years.

Furthermore, beyond retinopathy, prolonged HCQ usage can result in vortex keratopathy [80]. Keratopathy associated with chloroquine use could be assessed as a key risk factor and indicator of chloroquine retinopathy [39] as mentioned in the study by Paim-Marques of 16 vortex keratopathy cases in a large cohort of JSLE patients.

### Quality assessment of included studies

The risk of bias was ranked as high when the study reached up to 49% of “yes” scores, moderate when the study reached from 50 to 69% of “yes” scores, and low when the study reached more than 70% of “yes” scores in the JBI questionnaires [81].

Regarding case reports, JBI’s critical appraisal scores ranged from 37.5% to 100% (% yes), indicating high risk of bias for two studies [70, 78] due to limited clarity in patients’ history and diagnostic assessments (Appendix C, Tables 5-7)*.* Regarding cohorts, three studies indicated high risk of bias [74–76] (Appendix C, Table 8) due to no confounding factors’ clear identification and inadequate follow-up time reported, seemingly more like cross-sectional structure. Also, although the study of Tone et al. [28] was categorized as a cohort study, it exhibits traits more akin to a cross-sectional approach. Furthermore, its findings did not directly pertain to ocular manifestations but rather focused on the signs of dry eye in children with JSLE. Thus, it was not included in the summary of results of the cohorts. The critical appraisal of quality of the cross-sectional studies showed low risk of bias for all included studies except one with moderate risk of bias (Appendix C, Table 9). Case series studies showed moderate and high risk of bias (Appendix C, Table 10) due to unclear reporting of the demographics and vague criteria for inclusion in the case series [36, 37].

## Discussion

This review highlights several key findings about ocular manifestations of JSLE and raises important considerations for clinical practice and future research.

Firstly, the diverse array of ocular manifestations associated with JSLE is underscored, pointing to the systemic nature of the disease, with the retina being particularly susceptible. Importantly, these manifestations can range from mild to severe, with some posing a significant threat to vision and overall quality of life.

Comparing ocular manifestations between adults and children with SLE reveals a similar spectrum of symptoms [29], albeit with a more frequent presentation in the paediatric population [82]. Children with JSLE tend to experience a higher incidence of major organ involvement and a more severe clinical course overall [83, 84]. This suggests a need for heightened vigilance among clinicians caring for paediatric lupus patients, particularly regarding ocular health.

The pathophysiology of ocular manifestations in JSLE remains inadequately understood, with limited studies addressing this aspect specifically in children. The aetiology of ocular manifestations in JSLE is multifactorial, involving both the disease process itself and the effects of medications commonly used in its management. This review highlights the importance of monitoring children with JSLE for ocular damage, particularly given the potential for irreversible blindness in some cases [36]. A significant proportion of studies have indicated that ocular symptoms serve as the initial indication of the onset of the disease. These manifestations were observed either independently or concurrently with systemic acute disease. Recent studies show retinal alterations even in cases without obvious retinopathy [85].

The notable frequency of ocular manifestations at disease onset raises inquiries regarding the necessity of implementing specific diagnostic criteria aimed at facilitating early detection of JSLE. Similarly, it initiates a discourse on whether the existing diagnostic criteria should be revised to accommodate ocular manifestations, regardless of their presentation status [86].

Standardized protocols for ophthalmological assessment, including regular screenings at diagnosis and subsequent intervals, are recommended to facilitate early detection and intervention. To the authors’ best knowledge, there are currently no established guidelines for regular ophthalmological evaluations in children with JSLE. Based on the findings of this review, we recommend performing such assessments in children with JSLE:

- At the time of diagnosis, an initial comprehensive ophthalmic evaluation should be performed in children diagnosed with JSLE to establish a baseline assessment of ocular health.
- Every 6 months, routine follow-up evaluations should be scheduled, regardless of the presence of ocular symptoms. Thus, early detection of subtle changes in ocular health can be detected and timely intervention can be achieved to prevent progression or complications of ocular involvement.
- In cases of severe or active JSLE with significant systemic involvement, more frequent ophthalmic assessments may be warranted to closely monitor for ocular complications or adverse effects of immunosuppressive medications.
- Upon any vision complaint, children with JSLE should undergo prompt ophthalmic evaluation if they experience any changes in vision or other ocular symptoms, such as pain, redness, photophobia, or floaters.
- Self-monitoring of monocular vision and patient/parent education about the ophthalmological manifestations of the disease should be advised.

However, it is essential to acknowledge the limitations of the existing literature on ocular manifestations in JSLE. Our review primarily relies on limited and heterogeneous studies, predominantly comprising case reports and observational studies. Thus, drawing conclusions regarding the prevalence and characteristics of ocular manifestations in JSLE among children is challenging. Future research endeavours should aim to address these gaps through well-designed prospective studies and systematic reviews.

## Conclusion

In conclusion, this review underscores the significant impact of JSLE on paediatric ocular health and emphasizes the need for enhanced awareness, standardized assessment protocols, and further research to improve our understanding and management of ocular manifestations in this vulnerable population. Detecting ocular manifestations in paediatric patients diagnosed with JSLE is crucial for timely intervention, continuous monitoring of disease activity, and prevention of vision impairment. Despite ocular manifestations not being explicitly considered in the JSLE classification criteria, they can nonetheless serve as crucial indicators, acting as early warning signs for potential complications or flare-ups of the disease. Further research is necessary to understand the underlying mechanisms and pathogenesis of ocular manifestations in JSLE.

## Supplementary information

Appendix A

Appendix B

Appendix C
