# Correction: Ocular manifestations of Juvenile Systemic Lupus Erythematosus: a systematic review

**DOI:** 10.1038/s41433-025-03944-6

**Published:** 2025-08-14

**Authors:** Anna Nikolaidou, Theodora Gianni, Athanasia Sandali, Panagiotis Toumasis, Konstantinos Benekos, Efthymia Tsina

**Affiliations:** 1 Institute for Ophthalmic Research, University of Tuebingen, Tuebingen, Germany; 2 School of Medicine, Faculty of Health Sciences, Aristotle University of Thessaloniki, Thessaloniki, Greece; 3 Postgraduate Program MSc Ocular Surgery, School of Medicine, Faculty of Health Sciences, Aristotle University of Thessaloniki, Thessaloniki, Greece; 4 Ophthalmology Department, University of Ioannina, Ioannina, Greece; 5 Pediatric Ophthalmology Department, Athens Medical Centre, Athens, Greece

**Keywords:** Immunological disorders, Scientific community

Correction to: *Eye* 10.1038/s41433-025-03664-x, published online 17 February 2025

The article “Ocular manifestations of Juvenile Systemic Lupus Erythematosus: a systematic review”, written by [Anna Nikolaidou, Theodora Gianni, Athanasia Sandali, Panagiotis Toumasis, Konstantinos Benekos and Efthymia Tsina5, was originally published under exclusive license to The Royal College of Ophthalmologists. As a result of the subsequent decision to publish the article under the open access model, the article’s copyright notice was changed on 02 July 2025 to © The Author(s) 2025 and the article is now distributed under a Creative Commons Attribution 4.0 International License, which permits use, sharing, adaptation, distribution and reproduction in any medium or format, as long as you give appropriate credit to the original author(s) and the source, provide a link to the Creative Commons licence, and indicate if changes were made

The images or other third party material in this article are included in the article’s Creative Commons licence, unless indicated otherwise in a credit line to the material. If material is not included in the article’s Creative Commons licence and your intended use is not permitted by statutory regulation or exceeds the permitted use, you will need to obtain permission directly from the copyright holder.

To view a copy of this licence, visit http://creativecommons.org/licenses/by/4.0/.

The original article has been updated.

