# Supplementary material for: Ocular manifestations of Juvenile Systemic Lupus Erythematosus: a systematic review
Source: Eye (Lond). 2025 Feb 17;39(6):1056–69. doi: 10.1038/s41433-025-03664-x (PMC11978895; doi:10.1038/s41433-025-03664-x)
Supplement: Supplementary file 2 — Appendix B [file 41433_2025_3664_MOESM2_ESM.pdf]

Appendix B.
The following search string was used for PubMed search on October 14th 2024.

((juvenile lupus) OR ((lupus) AND (children OR child OR pediatric)) OR (Juvenile Systemic Lupus Erythematosus) OR (childhood-onset systemic lupus erythematosus) OR (Pediatric Onset Systemic Lupus Erythematosus)) AND ((ocular manifestation) OR (ophthalmic symptom) OR (ocular symptom) OR (ophthalmologic abnormalities))

Translations of terms as given by PubMed advanced search:
juvenile: "juvenile"[All Fields] OR "juvenile's"[All Fields] OR "juveniles"[All Fields] OR "juvenility"[All Fields]
lupus: "lupus vulgaris"[MeSH Terms] OR ("lupus"[All Fields] AND "vulgaris"[All Fields]) OR "lupus vulgaris"[All Fields] OR "lupus"[All Fields] OR "lupus erythematosus, systemic"[MeSH Terms] OR ("lupus"[All Fields] AND "erythematosus"[All Fields] AND "systemic"[All Fields]) OR "systemic lupus erythematosus"[All Fields]
children: "child"[MeSH Terms] OR "child"[All Fields] OR "children"[All Fields] OR "child's"[All Fields] OR "children's"[All Fields] OR "childrens"[All Fields] OR "childs"[All Fields]
child: "child"[MeSH Terms] OR "child"[All Fields] OR "children"[All Fields] OR "child's"[All Fields] OR "children's"[All Fields] OR "childrens"[All Fields] OR "childs"[All Fields]
pediatric: "paediatrics"[All Fields] OR "pediatrics"[MeSH Terms] OR "pediatrics"[All Fields] OR "paediatric"[All Fields] OR "pediatric"[All Fields]
Juvenile: "juvenile"[All Fields] OR "juvenile's"[All Fields] OR "juveniles"[All Fields] OR "juvenility"[All Fields]
Systemic Lupus Erythematosus: "lupus erythematosus, systemic"[MeSH Terms] OR ("lupus"[All Fields] AND "erythematosus"[All Fields] AND "systemic"[All Fields]) OR "systemic lupus erythematosus"[All Fields] OR ("systemic"[All Fields] AND "lupus"[All Fields] AND "erythematosus"[All Fields])
systemic lupus erythematosus: "lupus erythematosus, systemic"[MeSH Terms] OR ("lupus"[All Fields] AND "erythematosus"[All Fields] AND "systemic"[All Fields]) OR "systemic lupus erythematosus"[All Fields] OR ("systemic"[All Fields] AND "lupus"[All Fields] AND "erythematosus"[All Fields])
ocular: "ocular"[All Fields] OR "oculars"[All Fields]
manifestation: "manifest"[All Fields] OR "manifestating"[All Fields] OR "manifestation"[All Fields] OR "manifestations"[All Fields] OR "manifested"[All Fields] OR "manifesting"[All Fields] OR "manifestion"[All Fields] OR "manifestions"[All Fields] OR "manifests"[All Fields]
ophthalmic: "eye"[MeSH Terms] OR "eye"[All Fields] OR "ophthalmic"[All Fields] OR "ophthalmically"[All Fields] OR "ophthalmics"[All Fields]

symptom: "diagnosis"[Subheading] OR "diagnosis"[All Fields] OR "symptoms"[All Fields] OR "diagnosis"[MeSH Terms] OR "symptom"[All Fields] OR "symptom's"[All Fields] OR "symptomes"[All Fields]

ophthalmologic abnormalities: "eye abnormalities"[MeSH Terms] OR ("eye"[All Fields] AND "abnormalities"[All Fields]) OR "eye abnormalities"[All Fields] OR ("ophthalmologic"[All Fields] AND "abnormalities"[All Fields]) OR "ophthalmologic abnormalities"[All Fields]
