# Supplementary material for: Ocular manifestations of Juvenile Systemic Lupus Erythematosus: a systematic review
Source: Eye (Lond). 2025 Feb 17;39(6):1056–69. doi: 10.1038/s41433-025-03664-x (PMC11978895; doi:10.1038/s41433-025-03664-x)
Supplement: Supplementary file 3 — Appendix C [file 41433_2025_3664_MOESM3_ESM.pdf]

## Table 5. Critical appraisal (JBI) of Case reports (1)

| JBI- Case reports Question | Dorronsoro et al. (2023) | Deaner et al. (2020) | Huang et al. (2020) | Guleria et al. (2014) | Jeon et al. (2021) | Alhassan et al. (2021) | Firl et al. (2020) | Abbas et al. (2021) | Fischer et al. (2016) | Ahmadieh et al. (1994) |
|---|---|---|---|---|---|---|---|---|---|---|
| 1.Were patient's demographic characteristics clearly described? | Unclear | Unclear | Yes | Unclear | Unclear | Unclear | Unclear | Yes | Yes | No |
| 2.Was the patient's history clearly described and presented as a timeline? | No | No | Yes | No | Yes | No | Yes | Yes | Yes | Yes |
| 3.Was the current clinical condition of the patient on presentation clearly described? | Yes | Yes | Yes | Yes | Yes | Unclear | Yes | Yes | Yes | Yes |
| 4.Were diagnostic tests or assessment methods and the results clearly described? | Yes | Yes | Yes | Yes | Yes | Yes | Yes | Yes | Yes | Yes |
| 5.Was the intervention(s) or treatment procedure(s) clearly described? | Yes | Yes | Unclear | Yes | Yes | Yes | Yes | Yes | Yes | Unclear |
| 6.Was the post-intervention clinical condition clearly described? | Yes | Yes | Yes | Yes | Unclear | Unclear | No | Yes | Unclear | Unclear |
| 7.Were adverse events (harms) or unanticipated events identified and described? | Yes | Yes | Yes | Yes | Yes | Yes | No | Yes | Yes | Unclear |
| 8.Does the case report provide takeaway lessons? | Yes | Yes | Yes | Yes | Yes | Yes | No | Yes | Yes | Yes |
| **% Yes** | **75%** | **75%** | **87.5%** | **75%** | **75%** | **50%** | **50%** | **100%** | **87.5%** | **50%** |
| **Risk of bias*** | **Low** | **Low** | **Low** | **Low** | **Low** | **Moderate** | **Moderate** | **Low** | **Low** | **Moderate** |

**JBI: Joanna Briggs Institute* is rendered as: **JBI: Joanna Briggs Institute*

| Table 6. JBI* Case reports critical appraisal (2) | | | | | | | | | | | |
|---|---|---|---|---|---|---|---|---|---|---|---|
| **JBI- Case reports Question** | **Wei et al. (2009)** | **Lane-wala et al. (2015)** | **Chan et al. (2000)** | **Georgako-poulos et al. (2015)** | **Mohsen et al. (2020)** | **Moreno et al. (2019)** | **Lu et al. (2021)** | **Parakh et al. (2023)** | **Hamill et al. (2019)** | **Hackett et al. (1974)** | **Zhang et al. (2017)** |
| 1.Were patient's demographic characteristics clearly described? | Unclear | Unclear | Yes | Unclear | Yes | Unclear | Yes | Unclear | Yes | Unclear | Unclear |
| 2.Was the patient's history clearly described and presented as a timeline? | Yes | Yes | Yes | Yes | Yes | Yes | Unclear | Yes | Yes | Unclear | Unclear |
| 3.Was the current clinical condition of the patient on presentation clearly described? | Yes | Yes | Yes | Unclear | Yes | Yes | Unclear | Yes | Yes | Yes | Yes |
| 4.Were diagnostic tests or assessment methods and the results clearly described? | Yes | Yes | Yes | Unclear | Yes | Unclear | No | Yes | Yes | Unclear | Yes |
| 5.Was the intervention(s) or treatment procedure(s) clearly described? | Yes | Unclear | Unclear | Yes | Yes | Unclear | Yes | Yes | Unclear | Unclear | Yes |
| 6.Was the post-intervention clinical condition clearly described? | Yes | Yes | Yes | Yes | Yes | No | Unclear | Yes | Yes | Yes | Yes |
| 7.Were adverse events (harms) or unanticipated events identified and described? | Yes | Yes | Yes | Yes | Yes | Yes | Unclear | Yes | Yes | Unclear | Yes |
| 8.Does the case report provide takeaway lessons? | Yes | Yes | Yes | Yes | Yes | Yes | Yes | Yes | Yes | Yes | Yes |
| **% Yes** | **87.5%** | **75%** | **87.5%** | **62.5%** | **100%** | **50%** | **37.5%** | **87.5%** | **87.5%** | **37.5%** | **75%** |
| **Risk of bias** | **Low** | **Low** | **Low** | **Moderate** | **Low** | **Moderate** | **High** | **Low** | **Low** | **High** | **Low** |

**JBI: Joanna Briggs Institute*

| **Table 7. JBI* Case reports critical appraisal (3)** | | | | | | |
|---|---|---|---|---|---|---|
| **JBI- Case reports Question** | **Donnithorne et al. (2013)** | **Palkar et al. (2018)** | **Ho et al. (2008)** | **Parchand et al. (2016)** | **Graham et al. (1985)** | **Nguyen et al. (2000) - Case 3** |
| 1.Were patient's demographic characteristics clearly described? | Yes | Unclear | Unclear | Unclear | Yes | Yes |
| 2.Was the patient's history clearly described and presented as a timeline? | Yes | Yes | Yes | Yes | Yes | Yes |
| 3.Was the current clinical condition of the patient on presentation clearly described? | Yes | Yes | Yes | Yes | Yes | Yes |
| 4.Were diagnostic tests or assessment methods and the results clearly described? | Yes | Yes | Yes | Yes | Yes | Yes |
| 5.Was the intervention(s) or treatment procedure(s) clearly described? | Yes | Yes | Yes | Unclear | Unclear | Yes |
| 6.Was the post-intervention clinical condition clearly described? | Yes | Yes | Yes | Unclear | Yes | Yes |
| 7.Were adverse events (harms) or unanticipated events identified and described? | Yes | Yes | Yes | Yes | Yes | Yes |
| 8.Does the case report provide takeaway lessons? | Yes | Yes | Yes | Yes | Yes | Yes |
| **% Yes** | 100% | 87.5% | **87.5%** | 62.5% | 87.5% | 100% |
| **Risk of bias** | **Low** | **Low** | **Low** | **Medium** | **Low** | **Low** |
| **JBI: Joanna Briggs Institute* | | | | | | |

| Table 8. JBI* Case reports critical appraisal (4) | | | | | | | | | |
|---|---|---|---|---|---|---|---|---|---|
| **JBI Question** | **Kahwage et al. (2017)** | **Fraga et al. (2011)** | **Lim et al. (2020)** | **Tone et al. (2019)** | **Koutso-nikoli et al. (2015)** | **Salah et al. (2011)** | **Chan et al. (2016)** | **Ravelli et al. (2003)** | **Taddio et al. (2010)** |
| 1.Were the two groups similar and recruited from the same population? | Yes | N/A | N/A | Yes | N/A | N/A | Yes | N/A | Yes |
| 2.Were the exposures measured similarly to assign people to both exposed and unexposed groups? | Yes | N/A | Yes | N/A | N/A | N/A | Unclear | N/A | Yes |
| 3.Was the exposure measured in a valid and reliable way? | Yes | Yes | Yes | Yes | Yes | Yes | Yes | Yes | Yes |
| 4.Were confounding factors identified? | Yes | Yes | Unclear | Yes | Yes | Yes | No | Yes | Unclear |
| 5.Were strategies to deal with confounding factors stated? | Yes | Yes | No | Yes | Yes | Yes | No | Yes | No |
| 6.Were the groups/participants free of the outcome at the start of the study (or at the moment of exposure)? | Yes | Yes | Unclear | Yes | Unclear | Unclear | Unclear | Unclear | Unclear |
| 7.Were the outcomes measured in a valid and reliable way? | Yes | Unclear | Yes | Yes | Yes | Unclear | Yes | Yes | Yes |
| 8.Was the follow up time reported and sufficient to be long enough for outcomes to occur? | Yes | Yes | Yes | N/A | Yes | Yes | N/A | N/A | Yes |
| 9.Was follow up complete, and if not, were the reasons to loss to follow up described and explored? | Yes | Unclear | Unclear | N/A | Yes | Yes | N/A | N/A | Yes |
| 10.Were strategies to address incomplete follow up utilized? | Unclear | Unclear | Unclear | N/A | Yes | Yes | N/A | N/A | Yes |
| 11.Was appropriate statistical analysis used? | Yes | Yes | Yes | Yes | Yes | Yes | Yes | Yes | Yes |
| **% Yes** | **90.9%** | **54.5%** | **45.4%** | **63.6%** | **72.7%** | **63.6%** | **36.3%** | **45.4%** | **72.7%** |
| **Risk of Bias *** | **Low** | **Moderate** | **High** | **Moderate** | **Low** | **Moderate** | **High** | **High** | **Low** |

**JBI: Joanna Briggs Institute*

## Table 9. JBI* Cross-sectional studies critical appraisal

| JBI for cross-sectional studies question | Paim-Marques L. et al (2019) | Ağın et al (2019) | Gawdat G. et al. (2017) | Al-Mayouf et al (2003) |
|---|---|---|---|---|
| 1.Were the criteria for inclusion in the sample clearly defined? | Unclear | Yes | Yes | Yes |
| 2.Were the study subjects and the setting described in detail? | No | No | Yes | Yes |
| 3.Was the exposure measured in a valid and reliable way? | Yes | Yes | Yes | Yes |
| 4.Were objective, standard criteria used for measurement of the condition? | Yes | Yes | Yes | Yes |
| 5.Were confounding factors identified? | Yes | Yes | Yes | Yes |
| 6. Were strategies to deal with confounding factors stated? | No | Yes | Yes | Unclear |
| 7.Were the outcomes measured in a valid and reliable way? | Yes | Yes | Yes | Yes |
| 8.Was appropriate statistical analysis used? | Unclear | Yes | Yes | Yes |
| **%Yes** | **50%** | **87.5%** | **100%** | **87.5%** |
| **Risk of Bias** | **Moderate** | **Low** | **Low** | **Low** |

**JBI: Joanna Briggs Institute*

| Table 10. JBI* Case series studies Critical appraisal | | |
|---|---|---|
| **JBI for Case Series Question** | **Almeida et al. (2011)** | **Suri et al. (2015)** |
| 1. Were there clear criteria for inclusion in the case series? | Yes | No |
| 2.Was the condition measured in a standard, reliable way for all participants included in the case series? | Yes | Yes |
| 3.Were valid methods used for identification of the condition for all participants included in the case series? | Yes | Yes |
| 4.Did the case series have consecutive inclusion of participants? | Yes | No |
| 5.Did the case series have complete inclusion of participants? | Unclear | No |
| 6.Was there clear reporting of the demographics of the participants in the study? | Unclear | No |
| 7.Was there clear reporting of clinical information of the participants? | Yes | Yes |
| 8.Were the outcomes or follow up results of cases clearly reported? | Unclear | Yes |
| 9.Was there clear reporting of the presenting site(s)/clinic(s) demographic information? | Unclear | Unclear |
| 10.Was statistical analysis appropriate? | N/A | N/A |
| **%Yes** | **50%** | **40%** |
| **Risk of Bias*** | **Moderate** | **High** |
| **JBI: Joanna Briggs Institute* | | |
